# Acupuncture for rheumatoid vasculitis complicated by refractory foot ulcer: A case report

**DOI:** 10.1097/MD.0000000000035969

**Published:** 2023-11-10

**Authors:** Yaqing Cheng, Gang Cao, Qiaoli Yang, Shaohui Niu, Yina Fang, Chang Wang, Jianchun Cao

**Affiliations:** a Department of Peripheral Vascular, Dongfang Hospital of Beijing University of Chinese Medicine, Beijing, China; b Department of Peripheral Vasculoar, Xiyuan Hospital, China Academy of Chinese Medical Sciences, Beijing, China; c Department of General Surgery, Xi'an Hospital of Traditional Chinese Medicine, Shanxi, China

**Keywords:** acupuncture, case report, rheumatoid vasculitis, wound healing

## Abstract

**Introduction:**

Rheumatoid vasculitis (RV) is a frequently encountered complication of rheumatoid arthritis (RA), wherein skin vasculitis lesions are observed as a common clinical manifestation, encompassing skin purpura, erythema, vascular occlusion, ulcers, and gangrene. As a matter of fact, it marks the most severe extra-articular manifestation of RA. And the resultant ulcers tend to pose a greater challenge with regard to therapeutic interventions. We report a case of RV complicated by refractory foot ulcer that was successfully treated with puncture.

**Case presentation:**

A 62-year-old man with RV caused by RA developed refractory foot ulcers. Despite the application of topical antibiotics, the wound gradually expanded and remained unhealed for 7 months. Consequently, the patient sought an integrated therapeutic approach involving Traditional Chinese Medicine and was subsequently treated with acupuncture. After 12 weeks of acupuncture, the foot ulcers healed completely.

**Conclusion:**

Acupuncture has the potential to facilitate wound healing and may serve as a viable alternative treatment modality for wounds unresponsive to traditional therapeutic interventions.

## 1. Introduction

Rheumatoid vasculitis (RV) is a severe complication of rheumatoid arthritis (RA) that can lead to the formation of chronic and painful ulcers in the lower extremities.^[1,2]^ These ulcers are often resistant to treatment, can cause significant suffering in affected individuals.^[3]^ RV can affect any part of the body, but most commonly involve the skin, resulting in cutaneous vasculitis.^[4,5]^ Clinical manifestations of RV include purpura, erythema, papules, nodules, blisters, skin ulcers, livedo reticularis, Raynaud syndrome, gangrene, and discomfort such as numbness, pain, and fatigue in the lower extremities. Skin ulcers are particularly common and have a profound impact on the quality of life of the affected individuals.

RV management primarily relies on pharmaceutical interventions, particularly hormonal and immunosuppressant medications. Recently, biological agents, such as rituximab, tumor necrosis factor alpha inhibitors, and interleukin-6 inhibitors, have shown some therapeutic efficacy.^[6]^ However, the occurrence of limb breakdown remains inevitable, especially in patients who undergo prolonged steroid therapy. This therapy can lead to epidermal and dermal thinning, skin atrophy, alterations in wound healing, increased risk of infection, and recalcitrant ulcers. It is also a predisposing factor for ulcer development in patients with autoimmune disease.^[7]^

Acupuncture is an alternative therapeutic modality that has received increasing attention for its potential to promote wound healing. Recent research has demonstrated that acupuncture can accelerate wound healing and reduce the disease duration. The underlying mechanisms of this effect may involve the modulation of inflammation, promotion of angiogenesis, augmentation of fibroblast proliferation, stimulation of cell proliferation, and induction of extracellular matrix remodeling.^[8,9]^ Furthermore, the application of acupuncture around wounds can lead to reduced inflammation and enhanced microcirculation. In this report, we present a case of RV complicated by a refractory foot ulcer that was effectively treated with acupuncture, which resulted in complete wound healing and significant improvement in the skin color of the lower extremities.

## 2. Case presentation

### 2.1. Case

**Figure 1.**
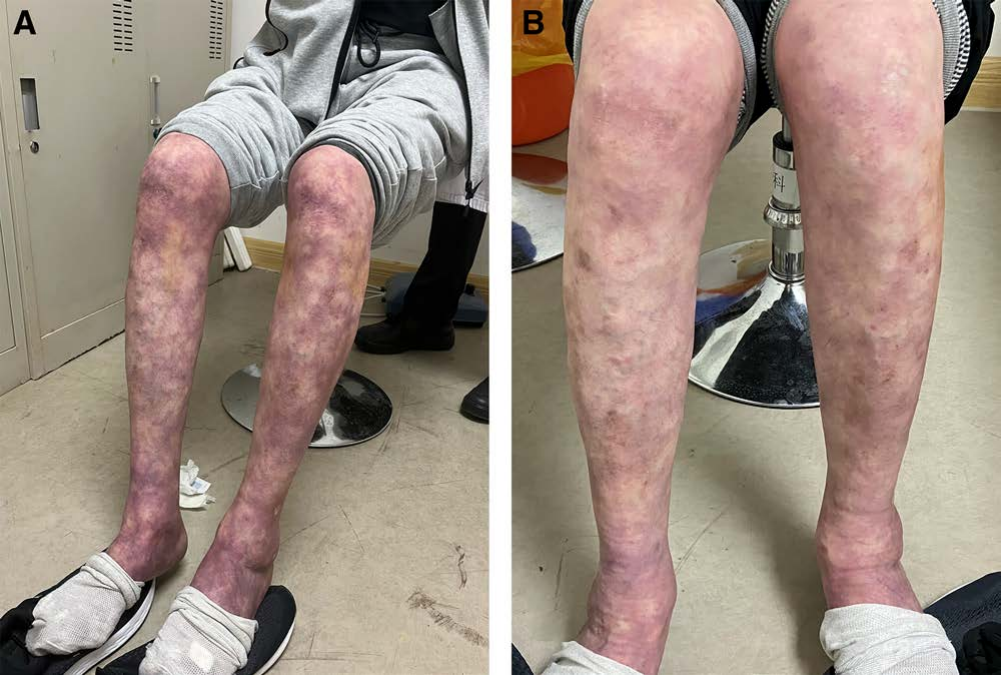
The livedo reticularis of lower extremities. (A) Prior to commencement of acupuncture therapy. (B) After undergoing acupuncture therapy.

A 62-year-old man who had experienced recurrent lower limb ulcers for a duration of 3 years visited our outpatient clinic on March 5, 2023. Despite these efforts, the foot ulcer did not progress for 7 months. The patient had a history of RA for 13 years, along with the use of leflunomide and prednisone acetate at dosages of 20 and 10 mg, respectively. Three years ago, the patient developed livedo reticularis on the skin of both lower limbs, and since then, has experienced cold and numb sensations in both lower limbs, as well as a sensation of walking on cotton with both feet. The patient suffered from recurrent lower-limb ulcers, which reversed healing within 1 to 2 months. Seven months prior to presentation, the patient developed a right-foot ulcer despite antibiotic treatment. The patient sought consultation because of pain in the wound, cold and numb sensations, and a sensation of stepping on cotton with both feet. The right heel showed a circular wound, which was tender to touch, and a severe presentation of reticular ecchymosis and purplish-red skin coloring. The patient healed from the previous ulcers on both lower limbs, leaving behind areas of pigment loss. Dorsalis pedis pulse perception in the foot was weakened (Figs. 1A and 3A). Serial laboratory examinations showed anti-cyclic citrullinated peptide antibody levels of >200 RU/mL, erythrocyte sedimentation rate of 65 mm/h, and rheumatoid factor of 502.52 IU/ML. Lower-extremity arterial ultrasonic Doppler: Lower-extremity arterial sclerosis with multiple plaque formation. Based on these findings, the provisional diagnosis was RV. Dermal biopsy was not possible because of certain constraints. The patient underwent a course of acupuncture from March 5 to May 28, 2023.

**Figure 2.**
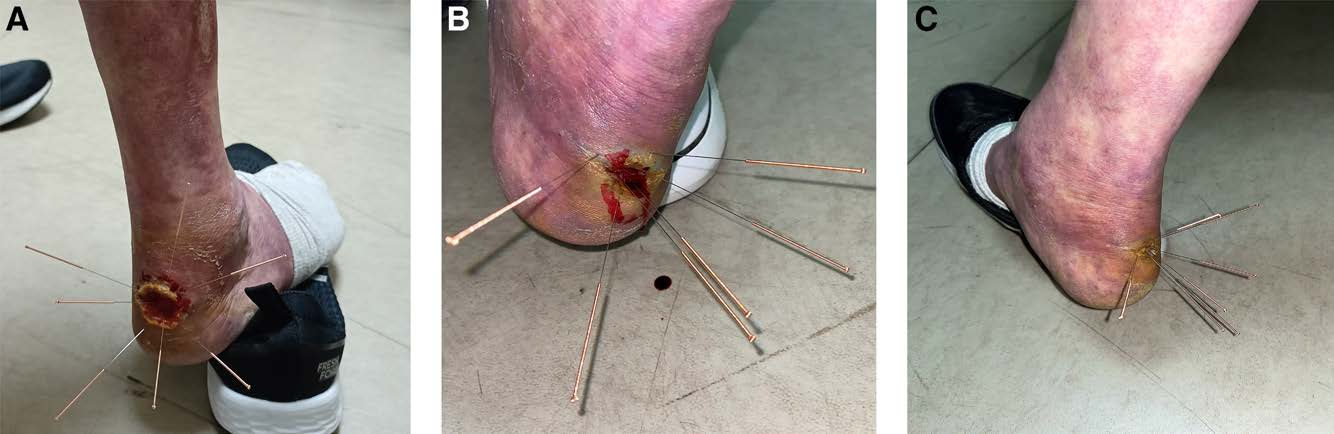
Acupuncture therapy for wound healing. (A–C) These pictures were taken from different angles.

**Figure 3.**
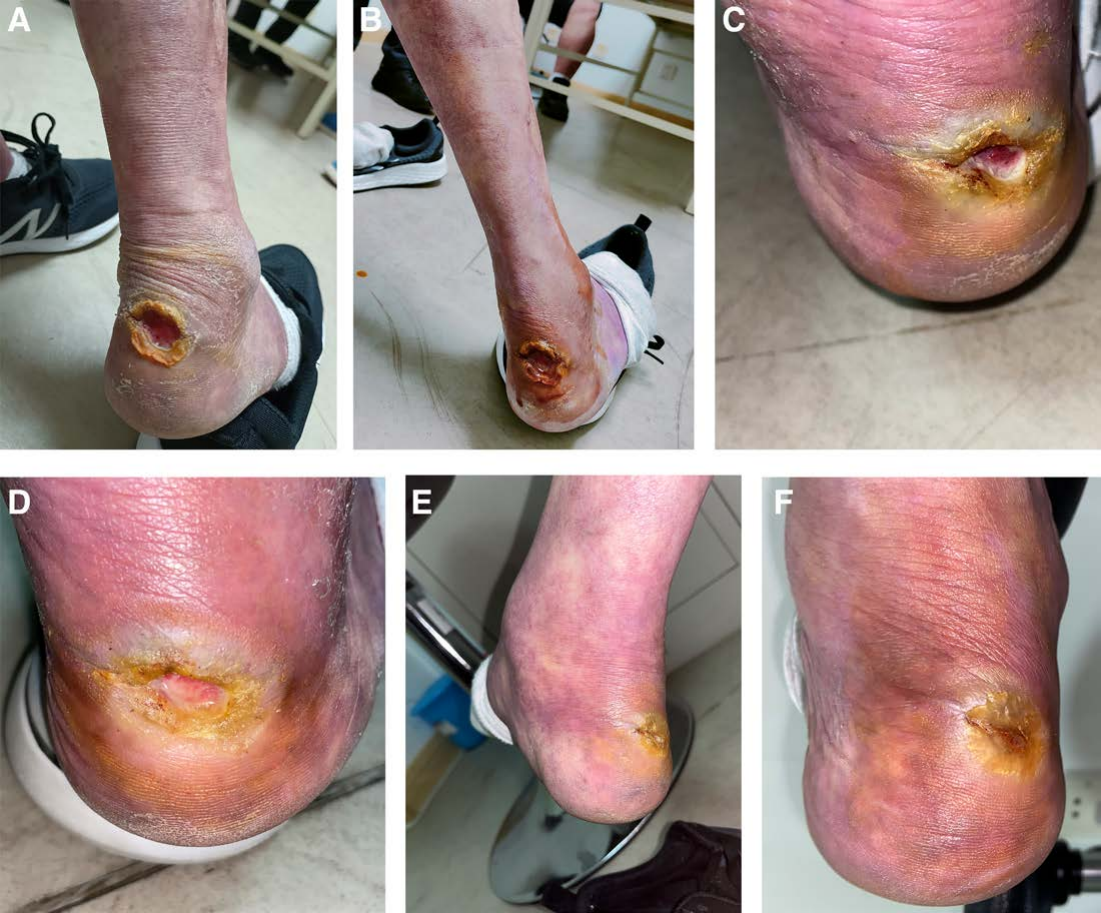
The wound healing process. (A) Before treatment. (B) Treatment for 1 week. (C) Treatment for 2 weeks. (D) Treatment for 4 weeks. (E) Treatment for 8 weeks. (F) Treatment for 12 weeks.

### 2.2. Treatment and intervention

Acupuncture was performed by puncturing the wound with a needle to a depth of approximately 2 mm. The needle aperture was not compressed, and hemorrhage spontaneously ceased. Subsequently, the needle was retained in the wound and the surrounding area was punctured. Acupuncture points were selected on both lower extremities including Zusanli, Yanglingquan, Shangjuxu, Xiajuxu, and Chengshan. The needles were left in place for 15 minutes after the needling sensation (Fig. 2A–C). Acupuncture was administered once a week in an outpatient setting. Following acupuncture, the wound was disinfected and dressed and normal saline solution was used for daily disinfection and dressing changes. After 1 week of treatment, the wound depth decreased by approximately 30%, and the wound area reduced by around 10%. Partial scab detachment was observed (Fig. 3B). By the end of the 2nd week, the wound depth decreased by approximately 50%, gradually healing from the edges towards the center. The wound area reduced by approximately 50%, and the scab detached (Fig. 3C). After 4 weeks of treatment, the wound depth and area decreased by approximately 80%, while pink granulation tissue filled the wound bed (Fig. 3D). After 8 weeks of treatment, the wound was nearing complete healing (Fig. 3E). After 12 weeks of treatment, the wound healed, the livedo reticularis improved, and the purple-red hue of both lower limbs significantly decreased (Figs. 1B and 3F).

## 3. Discussion

We report the case of a patient with refractory foot ulceration, a complication of the RV. The patient had a history of recurrent leg ulcers that typically resolved within 2 to 3 months. However, this foot ulcer persisted for 7 months despite treatment efforts. After acupuncture treatment, the wound healed, and the patient demonstrated a significant improvement in the skin coloration of the lower extremities. Our findings suggest that acupuncture may have a potential role in promoting wound healing and could be considered an alternative therapy for wound repair.

Currently, there is a lack of clinical trial data on the efficacy of acupuncture in wound treatment. However, animal experiments have suggested that acupuncture has the potential to mitigate pain, regulate inflammatory responses, and promote angiogenesis.^[10,11]^ Previous studies on acupuncture point selection have primarily focused on acupoints located around or distal to the wound on the lower limbs, with no known instances of direct needling of the wound.

In this particular case, we chose to needle around the wound to improve circulation and reduce inflammation, and directly apply the needles to the wound to release a small amount of blood, which can increase local blood flow and promote wound healing. Given the patient’s severe spider veins and purple-red skin color on the lower limbs, we selected specific acupoints, including Zusanli, Yanglingquan, Shangjuxu, Xiajuxu, and Chengshan. These acupoints were chosen to enhance muscle contraction strength, improve muscle pump function, and promote blood reflux to improve skin color.

In this particular case, needling around the wound enhance circulation, reduce inflammation, and directly apply the needles to the wound to encourage a slight amount of blood release. This wound augments local blood flow and promotes wound healing. Given the patient’s pronounced livedo reticularis and purplish-red skin pigmentation on the lower extremities, we targeted specific acupoints, including Zusanli, Yanglingquan, Shangjuxu, Xiajuxu, and Chengshan. These acupoints were chosen to improve muscle contraction strength, assist with muscle pump functionality, and promote blood reflux, ultimately resulting in a shift in the skin tone.

In this case, we attempted to use acupuncture on the wound, which has not been reported previously. Our initial findings suggested that wound healing was facilitated following local acupuncture, and we observed no deleterious effects on granulation tissue. These results suggest that acupuncture may significantly augment wound repair, representing a promising avenue for clinical application. However, this study has some potential limitations that should be acknowledged. First, due to the constraints of hospital conditions, a biopsy was not feasible, which hindered the ability to establish a definitive diagnosis. Second, the collection of case pictures lacked standardization, with inappropriate shooting angles and suboptimal picture quality. In addition, it is important to note that this study is a case report with inherent limitations in terms of its quality and generalizability. To ascertain the true efficacy of acupuncture in promoting wound healing, future high-quality randomized controlled trials are warranted. These future studies can provide more robust evidence and help validate the findings observed in this case report.

## 4. Summary and conclusion

Acupuncture has shown the potential to facilitate wound healing by increasing blood flow to the affected region, reducing inflammation, and promoting angiogenesis through various mechanisms. However, further investigations are needed to fully understand the efficacy and underlying mechanisms of acupuncture in expediting wound restoration.

## 5. Patient perspective

The efficacy of acupuncture therapy remains unclear. It successfully alleviated my discomfort, prompting my sense of regret for not having sought it sooner.

## Acknowledgments

The authors especially thank Professor Jianchun Cao as a chief physician who treated this patient.

## Author contributions

**Conceptualization:** Qiaoli Yang, Shaohui Niu.

**Visualization:** Yina Fang, Chang Wang.

**Writing—original draft:** Yaqing Cheng, Gang Cao.

**Writing—review & editing:** Jianchun Cao.
